# Constipation with megacolon or acute porphyria

**DOI:** 10.1093/gastro/goag026

**Published:** 2026-03-27

**Authors:** Junfu Chen, Shailan Zhou, Qiyi Chen, Hongliang Tian

**Affiliations:** Department of Functional Intestinal Diseases, General Surgery of Shanghai Tenth People's Hospital, Tenth People's Hospital of Tongji University, Shanghai 200072, P. R. China; School of Medicine, Tongji University, Shanghai 200072, P. R. China; Department of Functional Intestinal Diseases, General Surgery of Shanghai Tenth People's Hospital, Tenth People's Hospital of Tongji University, Shanghai 200072, P. R. China; The Nursing Department, Shanghai Tenth People's Hospital, Tenth People's Hospital of Tongji University, Shanghai 200072, P. R. China; Department of Functional Intestinal Diseases, General Surgery of Shanghai Tenth People's Hospital, Tenth People's Hospital of Tongji University, Shanghai 200072, P. R. China; School of Medicine, Tongji University, Shanghai 200072, P. R. China; Shanghai Gastrointestinal Microecology Research Center, Shanghai 200072, P. R. China; Shanghai Institution of Gut Microbiota Research and Engineering Development, Shanghai 200072, P. R. China; Department of Functional Intestinal Diseases, General Surgery of Shanghai Tenth People's Hospital, Tenth People's Hospital of Tongji University, Shanghai 200072, P. R. China; School of Medicine, Tongji University, Shanghai 200072, P. R. China; Shanghai Gastrointestinal Microecology Research Center, Shanghai 200072, P. R. China; Shanghai Institution of Gut Microbiota Research and Engineering Development, Shanghai 200072, P. R. China; Anhui University of Science and Technology First Clinical Medical College, Huainan, Anhui Province 232001, P. R. China; Bengbu First People's Hospital, Bengbu, Anhui Province 233000, P. R. China

## Introduction

Acute intermittent porphyria (AIP) is an autosomal dominant metabolic disorder caused by hydroxymethylbilane synthase (HMBS) deficiency, which leads to accumulation of neurotoxic porphyrin precursors and manifests as severe abdominal pain, gastrointestinal dysmotility and autonomic neuropathy, with a higher prevalence in women. The urine porphobilinogen test is the gold standard for diagnosing acute AIP attacks, yet its unavailability in some clinical settings poses great challenges for timely diagnosis, often requiring genetic testing as an alternative. Conventional treatments, including high-carbohydrate therapy and heme infusion, are effective for most patients, but clinical management of rare refractory gastrointestinal complications remains underexplored. Gastrointestinal dysmotility is common in AIP, while progression to megacolon requiring surgical intervention is exceedingly rare with limited clinical evidence.

## Case report

**Figure 1:**
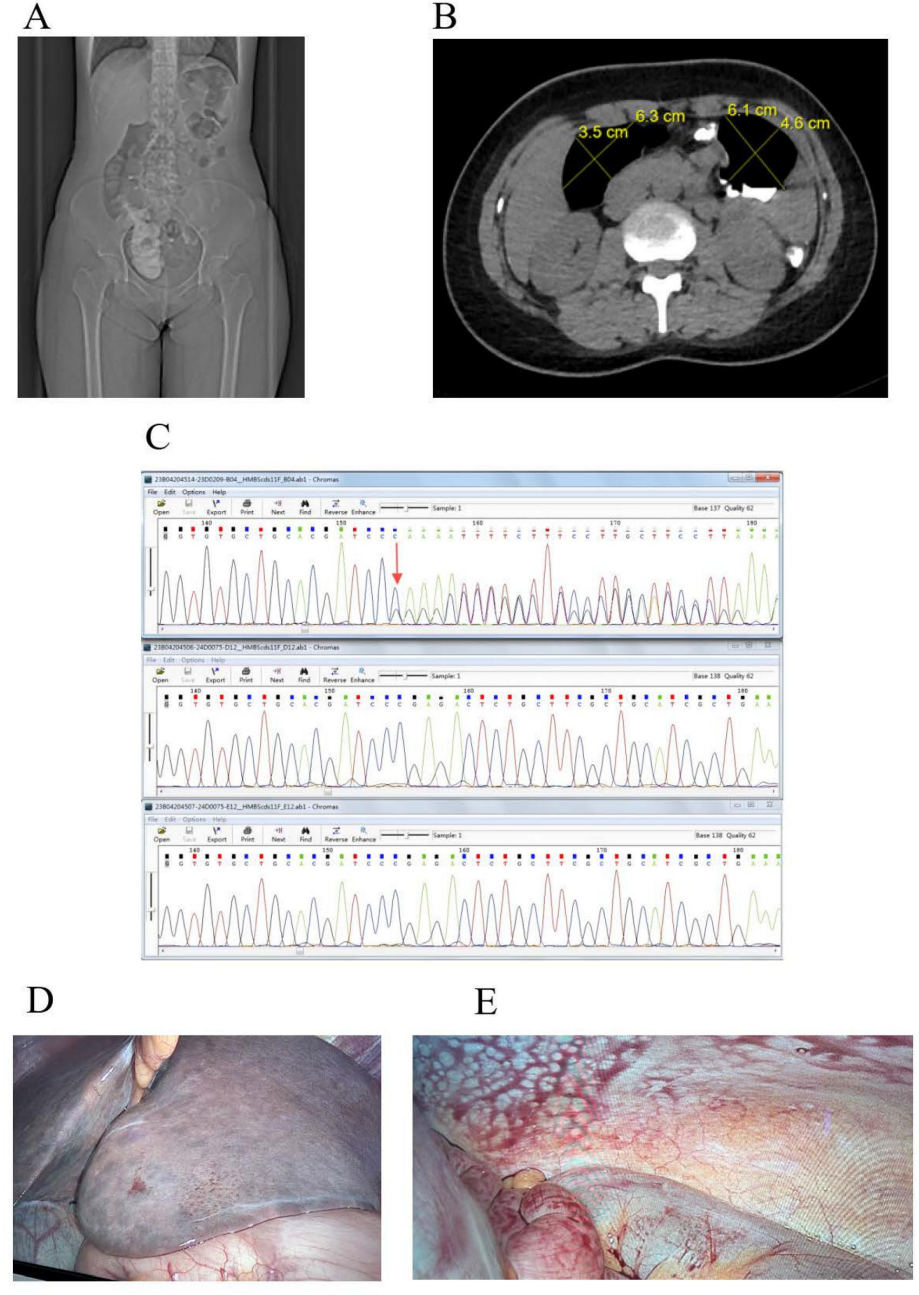
Imaging, genetic, and intraoperative features of acute intermittent porphyria complicated by megacolon. (A) Gastrointestinal contrast studies showing dilation of the right colon; (B) Preoperative CT scan demonstrating dilation of the right colon; (C) Sanger sequencing chromatograms of HMBS exon 11. Upper panel: index patient (proband) showing heterozygous c.723delC (p.Glu242Argfs*13). Middle panel: mother, wild-type sequence. Lower panel: father, wild-type sequence; (D) Intraoperative laparoscopic view showing a congested liver; (E): Congested bowel observed during surgery. AIP, acute intermittent porphyria; CT, computed tomography.

A 20-year-old Asian woman presented with a 6-month history of persistent upper abdominal pain that worsened over the past month, leading to hospital admission. For the preceding year, she had recurrent pain episodes linked to her menstrual cycle, initially raising suspicion of gynecological disorders such as dysmenorrhea or endometriosis. Gynecologic and neurologic evaluations were unremarkable. In the month before admission, her symptoms intensified with nausea, vomiting, and severe constipation, prompting transfer to our department. Laboratory investigations, including complete blood count and urinalysis, were within normal limits, whereas gastrointestinal contrast studies and abdominal CT demonstrated marked dilatation of the right colon (Fig. 1A and B).

A comprehensive differential diagnosis was systematically excluded through multidisciplinary evaluation. Acute peritonitis and bowel perforation were unlikely without peritoneal signs such as rebound tenderness, and CT revealed no free intraperitoneal air. Acute pancreatitis was ruled out by amylase and lipase levels and the absence of pancreatic abnormalities on imaging. Infectious colitis was excluded by normal inflammatory markers, negative stool cultures, and absence of diarrhea or hematochezia, while inflammatory bowel disease was unsupported by history or imaging. Toxic–metabolic and electrolyte disorders were excluded through normal biochemistry, and gynecological emergencies such as ectopic pregnancy or ovarian torsion were ruled out by negative pregnancy testing and normal pelvic ultrasonography. Severe abdominal pain with colonic dilation, disproportionate to imaging, raised suspicion for AIP. Detailed data of laboratory examinations are shown in Supplementary Table S1.

Unfortunately, although urine porphobilinogen test is regarded as the gold standard for confirming an acute attack of AIP, this assay was not available at our institution, nor could it be obtained from external laboratories at the time of presentation. In view of the patient’s urgent clinical condition and the need for prompt surgical decision-making, genetic testing was performed. Critical for AIP diagnosis and familial screening, this testing revealed a heterozygous frameshift mutation in HMBS exon 11 (HMBS (NM_00190.3) c.723delC (p.Glu242Argfs*13); Fig. 1C), confirming AIP. Though AIP is typically autosomal dominant, parental genetic analysis showed wild-type alleles, indicating a de novo mutation in the proband. This highlights that AIP may stem from spontaneous rather than only inherited mutations.

In line with clinical practice guidelines, the patient started on a high-carbohydrate diet, fluid supplementation, and analgesics. As acute porphyric attack lacked biochemical confirmation and no neurological signs (altered mental status, seizures), heme infusion was withheld. Despite days of supportive care, her condition failed to improve—abdominal pain intensified, with nausea, severe constipation, and progressive distension. A slow colonic transit study was conducted and yielded a positive result, consistent with markedly delayed intestinal transit. The patient underwent a colonic transit test, taking 1 capsule containing 20 radio-opaque markers orally. The markers passed through the intestinal tract naturally with food; abdominal X-ray films were taken at 24, 48, and 72 hours after administration to observe their distribution and excretion speed. The patient’s 72-hour marker residual rate was >20% (diagnostic cutoff value), and the test was judged positive. The patient suffered from intractable constipation accompanied by severe abdominal pain, which caused significant distress and was difficult to endure. The relapse was refractory to symptomatic management, necessitating surgical intervention. A subtotal colectomy with ascending–sigmoid anastomosis was performed. Intraoperative findings included hepatic congestion, marked colonic dilation, and bowel wall vascular congestion (Fig. 1D and E). Hepatic congestion might be associated with marked colonic dilatation, venous return obstruction, and the systemic stress response induced by the acute clinical episode. Histopathological exam of the resected colon revealed mucosal atrophy and submucosal venous vascular ectasia with congestion, consistent with chronic vascular compromise. Postoperatively, the patient had complete abdominal pain resolution and normal bowel function. High-carbohydrate nutrition was started, and at 4-month follow-up she remained symptom-free with no abdominal pain recurrence or other discomfort. Information of the drugs used is presented in Supplementary Table S2**.** 

## Discussion

Heme biosynthesis involves the conversion of succinyl-CoA and glycine to δ-aminolevulinic acid (δ-ALA) by 5-aminolevulinate synthase, followed by sequential enzymatic reactions that ultimately yield heme through the insertion of ferrous iron by ferrochelatase [1]. AIP is an autosomal dominant disorder caused by hydroxymethylbilane synthase (HMBS) deficiency, which may result in the accumulation of neurotoxic δ-ALA and porphobilinogen (PBG), leading to severe abdominal pain, neurovisceral dysfunction, and psychiatric symptoms [2]. AIP attacks are often triggered by hormonal fluctuations, medications, infections, or fasting, and are more prevalent in women [3]. Clinically, AIP presents with severe abdominal pain (more than 95% of Chinese patients [4]), gastrointestinal dysfunction, and autonomic neuropathy, accompanied by elevated urinary δ-ALA and PBG levels [5]. During acute attacks, elevated PBG levels can cause urine to appear red or dark red. The urine PBG test is the primary test to diagnose acute porphyria attacks. Genetic testing can be used in cases where urine PBG tests are not available.

Heme infusion is the preferred treatment for acute attacks in most settings. This therapy is typically well-tolerated and effective [1]. High carbohydrate therapy can also reduce porphyrin precursor accumulation and improve symptoms, though not as potent as hemin [6]. For patients with recurrent and severe attacks, liver transplantation has emerged as a final therapeutic option [7]. Another option includes givosiran, an RNA interference therapy that inhibits ALAS and prevents attacks. Other approaches currently in preclinical development include enzyme replacement therapy, gene therapy, and the regulation of protein homeostasis [1]. For symptomatic relief, mild pain can be managed with acetaminophen, while severe pain requires oral opioids initially, followed by intravenous administration if necessary, such as morphine [8].

For porphyria management, current guidelines do not recommend colectomy [9]. Analgesics for severe abdominal pain only give transient relief, not targeting the underlying cause. This patient with porphyria and severe constipation had intractable pain unresponsive to conventional therapy. Colectomy was performed for symptomatic megacolon with refractory severe constipation, rather than as standard treatment for AIP. Notably, the procedure resulted in complete and sustained resolution of abdominal pain, together with marked improvement in porphyria-related symptoms. Gastrointestinal dysmotility is a recognized manifestation of AIP, but progression to megacolon necessitating colectomy is exceedingly rare. In this case, histopathology revealed mucosal and muscular atrophy with vascular congestion and dilatation, findings consistent with chronic colonic dilatation but not specific to AIP. These observations suggest that autonomic dysfunction related to AIP may have contributed to the patient’s severe motility disorder, although a direct causal relationship cannot be established. Megacolon and severe constipation formed a self-perpetuating cycle that intensified abdominal pain and functional impairment. A slow colonic transit study confirmed markedly delayed transit. Many AIP patients exhibit constipation and mild colonic distension; one case has reported bowel ischemia during AIP attacks [10]. For porphyria patients with intractable constipation and megacolon unresponsive to conventional therapies, colectomy may be cautiously considered. Earlier diagnosis and timely hemin therapy might have prevented progression to severe megacolon and constipation. Based on the diagnostic challenges and clinical implications of the diseases highlighted in our study, we advocate that the findings presented herein be utilized as a plea for the establishment of proper diagnostic tools. These tools should specifically target all four types of acute porphyrias, tyrosinemia type 1, and lead intoxication, thereby facilitating early identification, reducing misdiagnosis rates, and ultimately optimizing the clinical management and prognosis of affected patients. For patients with suspected acute porphyria, the avoidance of porphyrogenic drugs is critical to clinical management and should be implemented promptly to prevent the worsening of clinical manifestations.

## Supplementary Material

goag026_Supplementary_Data
