# Supplementary material for: Constipation with megacolon or acute porphyria
Source: Gastroenterol Rep (Oxf). 2026 Mar 27;14:goag026. doi: 10.1093/gastro/goag026 (PMC13025055; doi:10.1093/gastro/goag026)
Supplement: goag026_Supplementary_Data [file goag026_supplementary_data.zip › 20-Mar-2026_125830_Supplementary_Tables.docx]

Supplementary Table S1: Detailed data of laboratory examinations.

| **Laboratory test** | **Result** | **Reference Range** |
| --- | --- | --- |
| Routine Blood Examination | | |
| C-Reactive Protein (CRP) | < 0.50 mg/L | < 8.20 mg/L |
| White Blood Cell (WBC) | 10.78 ×10^9/L | 3.5–9.5×10^9/L |
| Red Blood Cell (RBC) | 4.51 ×10^12/L | 3.8–5.1×10^12/L |
| Hemoglobin (Hb) | 131 g/L | 115–150 g/L |
| Hematocrit (HCT) | 38.1 % | 35–45% |
| Mean Corpuscular Volume (MCV) | 84.6 fL | 82–100 fL |
| Mean Corpuscular Hemoglobin (MCH) | 29.1 pg | 27–34 pg |
| Mean Corpuscular Hemoglobin Concentration (MCHC) | 344 g/L | 316–354 g/L |
| Platelet (PLT) | 131 ×10^9/L | 125–350×10^9/L |
| Neutrophil % | 94.4 % | 40–75% |
| Lymphocyte % | 2.9 % | 20–50% |
| Monocyte % | 2.6 % | 3–10% |
| Eosinophil % | 0.0 % | 0.4–8% |
| Basophil % | 0.1 % | 0–1% |
| Neutrophil Count | 10.16 ×10^9/L | 1.8–6.3×10^9/L |
| Lymphocyte Count | 0.32 ×10^9/L | 1.1–3.2×10^9/L |
| Monocyte Count | 0.29 ×10^9/L | 0.1–0.6×10^9/L |
| Eosinophil Count | 0.00 ×10^9/L | 0.02–0.52×10^9/L |
| Basophil Count | 0.01 ×10^9/L | 0–0.06×10^9/L |
| Red Cell Distribution Width (RDW) | 12.70 % | 10–15% |
| Mean Platelet Volume (MPV) | 11.7 fL | 9.4–13.5 fL |
| Plateletcrit (PCT) | 0.15 % | 0.11–0.27% |
| Platelet Distribution Width (PDW) | 16.5 % | 9–18.1% |
| Urine Routine Examination | | |
| Color | Yellow | Yellow |
| Appearance | Clear | Clear |
| Specific Gravity | 1.020 | 1.003–1.030 |
| pH | 6.0 | Morning Urine: 5.5–6.5 |
| Leukocyte Esterase | Negative (-) | Negative |
| Nitrite | Negative (-) | Negative |
| Protein | ± | Negative |
| Urine Glucose | Negative (-) | Negative |
| Ketone Bodies | 2+ | Negative |
| Urobilinogen | 1+ | (-) - (±) |
| Bilirubin | Negative (-) | Negative |
| Occult Blood | 3+ | Negative |
| Red Blood Cell (Microscopic) | 899/μl | Female: 0–7.5/μl |
| White Blood Cell (Microscopic) | 13/μl | Female: 0–12/μl |
| Epithelial Cell | 0 | Female: 1.5–26/μl |
| Cast | 0.0 | 0–1/μl |
| Crystal | 1.0 | 0–10/μl |

Supplementary Table S2: Information of the drugs used.

| Drug Name | Dosage | Dosage Form |
| --- | --- | --- |
| Sodium Lactate Ringer’s Injection | 500ml | st intravenous drip; qd intravenous drip |
| Potassium Chloride Injection + 10% Dextrose Injection | 1g + 500ml | st intravenous drip; qd intravenous drip |
| Somatostatin for Injection | 3mg (in 0.9% Sodium Chloride Injection 60ml) | st microinfusion pump |
| Compound Sodium Lactate and Glucose Injection | 500ml | st intravenous drip; qd intravenous drip |
| Cefoxitin Sodium for Injection | 1g (in 0.9% Sodium Chloride Injection 100ml) | st intravenous drip |
| Tramadol Hydrochloride Injection | 0.1g (in 0.9% Sodium Chloride Injection 100ml) st intravenous drip; 0.1g (in 100ml) q8h intravenous drip; 0.3g (in 0.9% Sodium Chloride Injection 50ml) st microinfusion pump | - |
| Anisodamine Hydrobromide Injection | 10mg | st intramuscular injection |
| 0.9% Sodium Chloride Injection | 12000ml st irrigation; 100ml + 0.1g st intravenous drip; 100ml + 0.1g q8h intravenous drip | - |
| Glucose and Sodium Chloride Injection + Compound Potassium Hydrogen Phosphate Injection | 500ml + 4ml | qd intravenous drip; st intravenous drip |
| Compound Amino Acid Injection (18AA-V) | 250ml | qd intravenous drip; st intravenous drip |
| Octreotide Acetate Injection | 0.1mg | q12h subcutaneous injection; st subcutaneous injection |
| 5% Dextrose Injection + Phloroglucinol Injection / Phloroglucinol Injection | 100ml + 40mg / 40mg | st intravenous drip |
| 10% Dextrose Injection | 500ml; 250ml | st intravenous drip |
| Dyclonine Hydrochloride Mucilage | 0.1g | st oral |
| Metoclopramide Hydrochloride Injection | 10mg | st intramuscular injection |
| Fuma Nasal Drops | 10ml | st nasal drops |
| Enteral Nutritional Suspension (Kangquangan) | 500ml | qd nasal feeding; st nasal feeding |
| Omeprazole Sodium for Injection | 40mg (in 0.9% Sodium Chloride Injection 100ml) | bid intravenous drip; st intravenous drip |
| Lactulose Oral Solution | 200ml | st oral |
| Lidocaine Hydrochloride Injection | 0.1g | st local anesthesia |
| Enteral Nutritional Powder | 400g | st oral |
