# Supplementary material for: Constipation with megacolon or acute porphyria
Source: Gastroenterol Rep (Oxf). 2026 Mar 27;14:goag026. doi: 10.1093/gastro/goag026 (PMC13025055; doi:10.1093/gastro/goag026)
Supplement: goag026_Supplementary_Data [file goag026_supplementary_data.zip › Supplementary_Table1.zip!/Supplementary_Table 1.docx]

**Supplementary Table 1.** Information of the drugs used.

| Drug Name | Dosage | Dosage Form |
| --- | --- | --- |
| Sodium Lactate Ringer’s Injection | 500ml | st intravenous drip; qd intravenous drip |
| Potassium Chloride Injection + 10% Dextrose Injection | 1g + 500ml | st intravenous drip; qd intravenous drip |
| Somatostatin for Injection | 3mg (in 0.9% Sodium Chloride Injection 60ml) | st microinfusion pump |
| Compound Sodium Lactate and Glucose Injection | 500ml | st intravenous drip; qd intravenous drip |
| Cefoxitin Sodium for Injection | 1g (in 0.9% Sodium Chloride Injection 100ml) | st intravenous drip |
| Tramadol Hydrochloride Injection | 0.1g (in 0.9% Sodium Chloride Injection 100ml) st intravenous drip; 0.1g (in 100ml) q8h intravenous drip; 0.3g (in 0.9% Sodium Chloride Injection 50ml) st microinfusion pump | - |
| Anisodamine Hydrobromide Injection | 10mg | st intramuscular injection |
| 0.9% Sodium Chloride Injection | 12000ml st irrigation; 100ml + 0.1g st intravenous drip; 100ml + 0.1g q8h intravenous drip | - |
| Glucose and Sodium Chloride Injection + Compound Potassium Hydrogen Phosphate Injection | 500ml + 4ml | qd intravenous drip; st intravenous drip |
| Compound Amino Acid Injection (18AA-V) | 250ml | qd intravenous drip; st intravenous drip |
| Octreotide Acetate Injection | 0.1mg | q12h subcutaneous injection; st subcutaneous injection |
| 5% Dextrose Injection + Phloroglucinol Injection / Phloroglucinol Injection | 100ml + 40mg / 40mg | st intravenous drip |
| 10% Dextrose Injection | 500ml; 250ml | st intravenous drip |
| Dyclonine Hydrochloride Mucilage | 0.1g | st oral |
| Metoclopramide Hydrochloride Injection | 10mg | st intramuscular injection |
| Fuma Nasal Drops | 10ml | st nasal drops |
| Enteral Nutritional Suspension (Kangquangan) | 500ml | qd nasal feeding; st nasal feeding |
| Omeprazole Sodium for Injection | 40mg (in 0.9% Sodium Chloride Injection 100ml) | bid intravenous drip; st intravenous drip |
| Lactulose Oral Solution | 200ml | st oral |
| Lidocaine Hydrochloride Injection | 0.1g | st local anesthesia |
| Enteral Nutritional Powder | 400g | st oral |
